# Surgical treatment of recalcitrant gastroesophageal reflux disease in patients with systemic sclerosis: a systematic review

**DOI:** 10.1007/s00423-021-02118-8

**Published:** 2021-02-21

**Authors:** Alberto Aiolfi, Mario Nosotti, Kazuhide Matsushima, Carolina Perali, Cristina Ogliari, Nicoletta Del Papa, Gianluca Bonitta, Davide Bona

**Affiliations:** 1 Department of Biomedical Science for Health, Division of General Surgery, University of Milan, Istitituto Clinico Sant’Ambrogio, Via Luigi Giuseppe Faravelli, 16, 20149 Milan, Italy; 2 Department of Pathophysiology and Transplantation, Thoracic Surgery and Lung Transplant Unit Fondazione IRCCS Ca’ Granda - Ospedale Maggiore Policlinico, Milan, Italy; 3 Division of Acute Care Surgery, LAC+USC Medical Center, University of Southern California, 2051 Marengo Street, IPT, C5L100, Los Angeles, CA 90033 USA; 4 Department of Rheumatology, UOC Day Hospital of Rheumatology, ASST G. Pini-CTO, 20122 Milan, Italy

**Keywords:** Systemic sclerosis, Gastroesophageal reflux, Fundoplication, Roux en-Y gastric bypass

## Abstract

**Introduction:**

Gastroesophageal reflux disease (GERD) is frequently seen in patients with systemic sclerosis (SSc). Long-standing GERD may cause esophagitis, long-segment strictures, and Barrett’s esophagus and may worsen pre-existing pulmonary fibrosis with an increased risk of end-stage lung disease. Surgical treatment of recalcitrant GERD remains controversial. The purpose of this systematic review was to summarize the current data on surgical treatment of recalcitrant GERD in SSc patients.

**Materials and methods:**

A systematic literature review according to PRISMA and MOOSE guidelines. PubMed, EMBASE, and Web of Science databases were consulted.

**Results:**

A total of 101 patients were included from 7 studies. The age ranged from 34 to 61 years and the majority were females (73.5%). Commonly reported symptoms were heartburn (92%), regurgitation (77%), and dysphagia (74%). Concurrent pulmonary disease was diagnosed in 58% of patients. Overall, 63 patients (62.4%) underwent open fundoplication, 17 (16.8%) laparoscopic fundoplication, 15 (14.9%) Roux en-Y gastric bypass (RYGB), and 6 (5.9%) esophagectomy. The postoperative follow-up ranged from 12 to 65 months. Recurrent symptoms were described in up to 70% and 30% of patients undergoing fundoplication and RYGB, respectively. Various symptoms were reported postoperatively depending on the type of surgical procedures, anatomy of the valve, need for esophageal lengthening, and follow-up.

**Conclusions:**

The treatment of recalcitrant GERD in SSc patients is challenging. Esophagectomy should be reserved to selected patients. Minimally invasive RYGB appears feasible and safe with promising preliminary short-term results. Current evidence is scarce while a definitive indication about the most appropriate surgical treatment is lacking.

**Supplementary Information:**

The online version contains supplementary material available at 10.1007/s00423-021-02118-8.

## Introduction

Systemic sclerosis (SSc) is a rare autoimmune disease characterized by fibrosis of small arteries and anomalous deposition of collagen in the skin, lungs, and gastrointestinal tract [1]. The esophagus is frequently involved (up to 90% of patients), and up to 80% of patients with SSc may suffer from gastroesophageal reflux disease (GERD) [2, 3]. The etiology of GERD in these patients is likely to be multifactorial and may derive from a combination of impaired saliva production, aperistaltic esophagus, incompetent lower esophageal sphincter (LES), and gastroparesis [4]. Complications related to long-standing GERD include esophagitis, long-segment strictures, Barrett’s esophagus, and recurrent aspiration pneumonitis with consequent pulmonary fibrosis [5]. These conditions, in combination with the pulmonary manifestations of SSc, may increase the risk of end-stage lung disease requiring lung transplantation [6].

Treatment of GERD in patients with SSc ranges from medical to surgical interventions and often depends upon the time of diagnosis and comorbid conditions. Twice-daily proton pump inhibitor (PPI) therapy has been shown to be effective; however, up to 40% of patients are non-responders to PPI treatment [7]. Surgical procedures have been proposed to reduce GERD symptoms and improve patients’ quality of life. However, the application of surgical treatments in the setting of SSc poses significant challenges given the associated esophageal dysmotility disorders [8]. The purpose of this systematic review was to summarize the current knowledge on surgical treatment of recalcitrant GERD in patients with SSc.

## Materials and methods

**Fig. 1:**
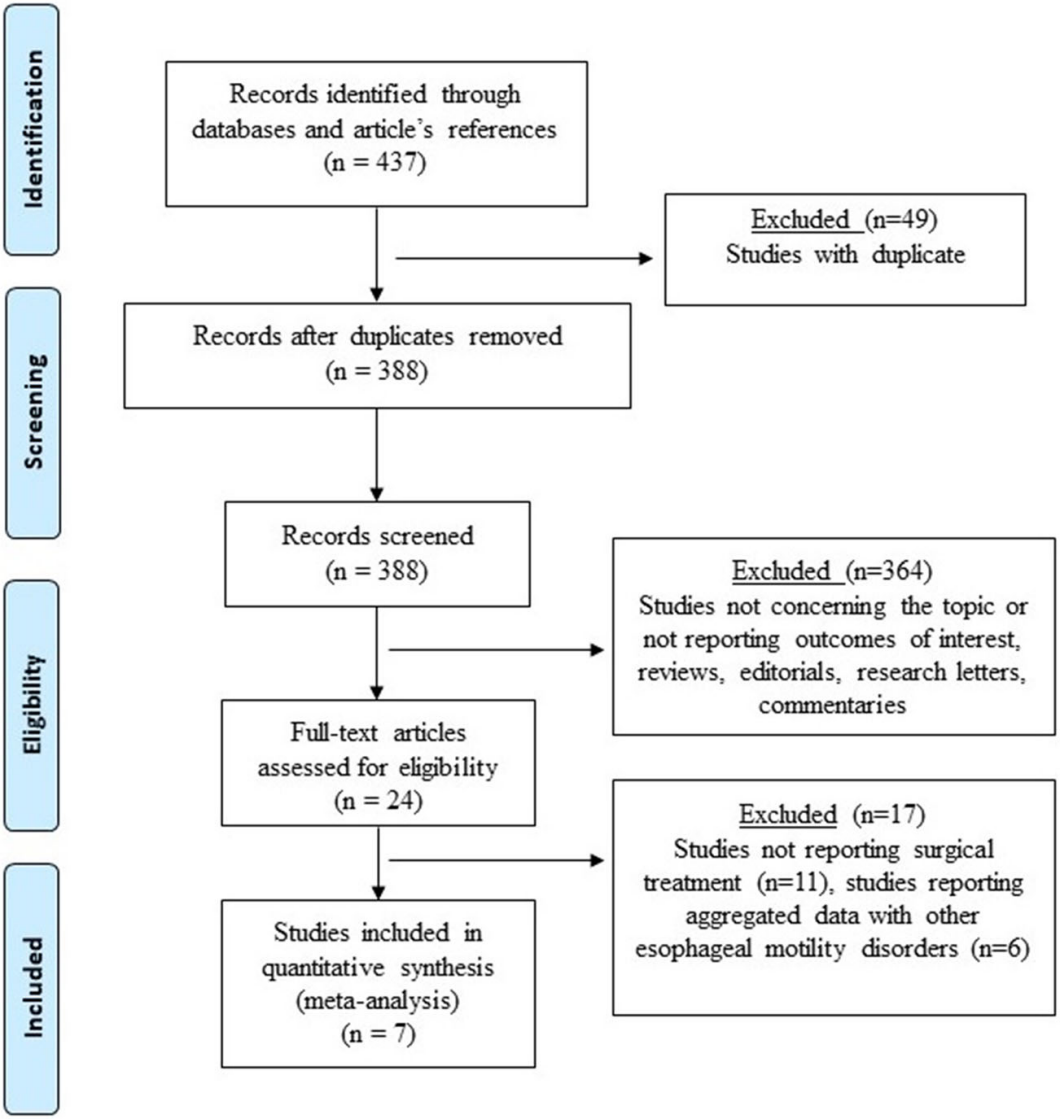
The Preferred Reporting Items for Systematic Reviews and Meta-Analyses (PRISMA) checklist diagram

A systematic review was performed according to the guidelines from the Preferred Reporting Items for Systematic Reviews and Meta-Analyses checklist (PRISMA) and Meta-analyses of Observation Studies in Epidemiology (https://www.editorialmanager.com/jognn/account/MOOSE.pdf) [9] (Fig. 1). Institutional review board approval was not required. An extensive literature review was performed to identify all English-written published articles on the surgical management of GERD in patients with SSc. PubMed, EMBASE, and Web of Science databases were consulted [10] matching the terms “GERD,” “anti-reflux surgery,” “gastroesophageal reflux disease,” “surgical management,” “fundoplication,” “systemic sclerosis,” “esophageal,” “scleroderma,” “esophagectomy,” and “gastric bypass” with “AND” and “OR” until 30 January 2020 (Appendix). The search was completed by consulting the listed references of each article.

### Eligibility criteria

Inclusion criteria are as follows: (a) articles reporting outcomes for the treatment of recalcitrant GERD in SSc patients; (b) English-written studies; (c) articles with the longest follow-up or the largest sample size when two or more papers were published by the same institution and study group or used the same dataset. Exclusion criteria are as follows: (a) non-English written articles; (b) studies without clear methodology; (c) reports of anti-reflux surgery in patients not affected by systemic sclerosis or in patients affected by dysmotility disorders other than SSc.

### Data extraction

Three authors (CP, AA, CO) independently extracted data from eligible studies. Data extracted included study characteristics (first author name, year, and journal of publication), number of patients included in the series, time frame, clinical and demographic characteristics of patients’ population, type of surgical procedure, and outcomes. A fourth author (DB) clarified disagreements.

### Quality evaluation

The quality of observational studies was independently assessed by three authors (AA, CP, GB) with the Risk of Bias In Non-Randomized Studies (ROBINS-I) instrument. Confounding, selection, classification, intervention, missing data, outcomes measurement, and reporting bias were considered. Each domain was estimated with “yes,” “probably yes,” “probably no,” or “no,” and studies were categorized as having low, moderate, serious, or critical risk of bias [11].

## Results

### Literature search and study quality

Seven articles published between 1981 and 2020 were found and included in the systematic review. Five articles were retrospective single-center case series while two were case reports (Table 1). According to the ROBINS-I tool, three studies were categorized as having moderate risk of bias while four were categorized as having severe risk of bias. Outcomes might have been influenced by confounding and selection bias because inclusion/exclusion criteria and patient treatment allocation were heterogeneous among studies (Supplementary Table 1).

**Table 1:** Demographic, clinical, and operative data. *Ret* retrospective, *CR* case report, *RYGB* Roux en-Y gastric bypass, *nr* not reported. Data are reported as numbers, mean ± standard deviation, and median (range)

Author, year	Study period	Study design	No. of patients	Age	Sex, female (no.)	Mean BMI (kg/m^2^)	Symptoms duration (years)	Surgical approach	Follow-up (months)
Orringer et al., 1981 [12]	nr	Ret, single center	37	nr	nr	nr	nr	Collis-Nissen (*n* = 20), Collis-Belsey (*n* = 17)	22–42
Mansour et al., 1988 [13]	1975–1987	Ret, single center	11	24–65	nr	nr	3.6	Belsey (*n* = 6), Collis-Belsey (*n* = 2), Nissen (*n* = 2), Collis-Nissen (*n* = 1)	24–144
Poirer et al., 1994 [14]	nr	Ret, single center	14	34–60	11	59 (kg)	7	Nissen (*n* = 10), Collis-Nissen (*n* = 2), Collis-Belsey (*n* = 1), antrectomy, vagotomy, and R-Y reconstruction (*n* = 1)	65
Kent et al., 2007 [8]	1995–2007	Ret, single center	23	61 (30–75)	16	RYGB: 32.3 Fundo: nr	nr	Laparoscopic RYGB (*n* = 8), laparoscopic Nissen (*n* = 5), laparoscopic Collis-Nissen (*n* = 3), laparoscopic Collis-Toupet (*n* = 1), Laparoscopic Toupet (*n* = 1), minimally invasive esophagectomy (*n* = 3), open transhiatal esophagectomy (*n* = 2)	RYGB 13 (1–59) Fundo 36 (1–64) Esophagectomy 14 (8–18)
Yekeler et al., 2008 [15]	2008	CR	1	20	0	nr	2	Ivor-Lewis esophagectomy (laparotomy and right thoracotomy)	24
Andrade et al., 2017 [16]	2017	CR	1	44	1	nr	25	Robotic Dor	12
Yan et al., 2018 [17]	2004–2016	Ret, single center	14	54 (37–65)	11	RYGB: 28 ± 4 Fundo: 24 ± 5	nr	Laparoscopic RYGB (*n* = 7), laparoscopic Nissen (*n* = 2), laparoscopic Collis-Toupet (*n* = 2), laparoscopic Toupet (*n* = 1), open Toupet (*n* = 1), robotic Dor (*n* = 1)	RYGB 19 (1–164) Fundo 97 (28–204)

### Preoperative patients’ characteristics

A total of 101 SSc patients with recalcitrant GERD were included. The patient age ranged from 34 to 61 years and 73.5% were females. The preoperative body mass index (BMI) was reported in two articles and ranged from 19 to 32.3 kg/m^2^. None of the articles reported the preoperative American Society of Anesthesiologists (ASA) physical status, and comorbidities were reported in 4 articles. The duration of symptoms related to GERD ranged from 4 months to 25 years. Overall, 97% of patients were taking PPI or H2 blockers before the operation. Most commonly reported symptoms were heartburn (92%), regurgitation (77%), and dysphagia (74%) (Table 2). Esophagitis was diagnosed in 76% of patients, hiatal hernia in 63%, esophageal peptic stricture in 31%, and Barrett’s esophagus in 15%. The results of esophageal manometry study was reported in five articles; an absent esophageal body peristalsis was diagnosed in 83% of patients while 57% of patients had low basal LES pressure (< 10 mmHg). Preoperative 24-h pH study was reported in three studies. Abnormal acid exposures were noted in 90% of patients. Concurrent severe pulmonary disease or pulmonary fibrosis was diagnosed in 58% of patients. Of those, four patients received preoperative and three patients received postoperative transplant. Overall, 56% patients were taking immunosuppressant medications (e.g., corticosteroids, mycophenolate mofetil) at the time of surgery.

**Table 2:** Patients’ symptoms. Data are reported as percentages (%)

Symptoms	%
Heartburn	92
Regurgitation	77
Dysphagia	74
Esophagitis (grades A–D)	76
Barrett’s esophagus	15
Esophageal stricture	31
Hiatal hernia	63
Interstitial lung disease	58

### Surgical treatments

The list of surgical treatments is shown in Table 3. Esophagectomy was performed in 6 patients (5.9%). Open fundoplication, via laparotomy or thoracotomy, was performed in 63 patients. Common surgical techniques were Collis-Nissen (*n* = 23), Collis-Belsey (*n* = 20), and Nissen (*n* = 12) fundoplication (Table 3). Minimally invasive treatment was adopted in 32 patients with fundoplication (*n* = 17) and Roux en-Y gastric bypass (RYGB) (*n* = 15). Nissen (*n* = 7), Collis-Nissen (*n* = 3), and Collis-Toupet (*n* = 3) fundoplication were the most commonly adopted fundoplication. In the group of patients that underwent laparoscopic RYGB, twelve had < 100-cm Roux limbs while three had 150-cm Roux limbs.

**Table 3:** Summary of different surgical approaches in the population. *Robotic

Surgical approach	Treatment	No. of patients
Open (laparotomy or thoracotomy)	Collis-Nissen	23
	Collis-Belsey (240°)	20
	Nissen	12
	Belsey (240°)	6
	Transhiatal esophagectomy	2
	Ivor-Lewis esophagectomy	1
	Antrectomy with Roux-en Y reconstruction	1
	Toupet	1
Minimally invasive (laparoscopy)	RYGB	15
	Nissen	7
	Collis-Nissen	3
	Collis-Toupet	3
	Esophagectomy	3
	Toupet	2
	Dor*	2

### Perioperative complications

The overall postoperative complication rate following esophagectomy was 83%. Commonly reported complications included pneumonia (*n* = 3), anastomotic stricture (*n* = 2), and anastomotic leak (*n* = 1). Postoperative mortality was 17%. In the open fundoplication group, the reported postoperative morbidity and mortality rates were 6.3% and 1.6%, respectively. For minimally invasive treatments, the overall postoperative complication rate was 5.9% in the fundoplication group and 13.3% for RYGB. The overall postoperative mortality was 3.1% and was reported in one RYGB patient who experienced graft failure for recurrent aspiration pneumonia.

### Follow-up

The postoperative follow-up periods ranged from 12 to 65 months. Overall, three patients that underwent open partial or total fundoplication during their initial operation required esophagectomy for intractable GERD. One patient who had laparoscopic Nissen fundoplication required conversion to RYGB for recurrent dysphagia, and one patient with RYGB required diagnostic laparoscopy for nausea, vomiting, and dysphagia with no evidence of internal hernia. Peptic stricture improved or resolved in 64.5% of patients, 32.2% required repeated endoscopic dilation, and 3.3% required esophagectomy for recurrent stricture after laparoscopic Collis-Toupet fundoplication. Various postoperative complains were reported depending on different surgical procedures, anatomy of the valve, need for esophageal lengthening (Collis procedure), follow-up time, and articles reporting. In patients treated with fundoplication, recurrent symptoms such as dysphagia and heartburn were described in up to 70% of patients while in RYGB, recurrent symptoms were diagnosed in up to 30% of patients. A trend toward improved GERD Health-Related Quality of life (GERD-HRQL) was reported for RYGB compared to fundoplication (4.0 vs. 15.6; *p* = 0.05).

## Discussion

The treatment of GERD in the setting of SSc is challenging because of its multifactorial pathogenesis. Reduced salivary production, impaired esophageal motility, ineffective LES, and sometimes the presence of delayed gastric emptying lead to pathologic reflux refractory to medical therapy in up to 40% of patients [7, 8]. Different surgical options have been proposed in non-responders; however, due to different surgical indications and the rarity of the disease, a definitive and robust evidence on the best surgical approach is still lacking. In addition, given the frequent associated gastrointestinal dysmotility disorders and pulmonary impairment, an assessment of postoperative symptoms and outcomes is even more challenging. Choosing an appropriate surgical approach in these patients is of outmost importance to improve gastroesophageal reflux, dysphagia, and health-related quality of life, and for a potential benefit on idiopathic pulmonary fibrosis. Despite the complex disease mechanisms, lung protection provided by a well-performed anti-reflux surgery may be beneficial in preventing repeated lung injury from chronic acid exposure [18].

Esophagectomy has been described as a potential option but is associated with significant morbidity and mortality. In this systematic review, 6 patients underwent esophagectomy for the presence of fibrotic long-segment strictures recalcitrant to multiple endoscopic dilations. The postoperative complication, anastomotic leak/stricture, and mortality rates were notably high. In SSc patients, the incidence of anastomotic leak, anastomotic stricture, and complications related to the gastric conduit may be increased because of the microvascular disease, whereas pulmonary complications may be more frequent because of the pre-existing interstitial lung disease [8]. Therefore, the indication for esophagectomy should be carefully evaluated only in highly selected patients [19].

Open fundoplication via laparotomy or thoracotomy has been described with conflicting results. Orringer et al. reported a significant postoperative GERD improvement (32% vs. 100%) and dysphagia (38% vs. 81%) in 37 SSc patients at 34-month mean follow-up. Similarly, Poirer et al. reported persistent but significantly reduced pathologic acid exposures in 14 SSc patients assessed by 24-h pH testing. However, 71% of patients complained of recurrent postoperative dysphagia. By contrast, Mansour et al. reported unsatisfying outcomes with significant dysphagia (64%) and reflux rate (100%) in a series of 11 SSc patients undergoing open Belsey (*n* = 6), Collis-Belsey (*n* = 2), Nissen (*n* = 2), and Collis-Nissen (*n* = 1) fundoplication [13]. Because of the intractable GERD, 3 patients underwent esophagectomy with colonic interposition. The authors concluded that the temporary control of reflux and dysphagia can be achieved with anti-reflux procedures, but will ultimately fail over the course of years. Because of the different surgical approaches (laparotomy vs. thoracotomy), surgical techniques (total vs. partial fundoplication), and follow-up, a quantitative and robust analysis was impracticable. Furthermore, the choice of a full-wrap fundoplication is highly questionable in patients with esophageal motility disorders.

**Fig. 2:**
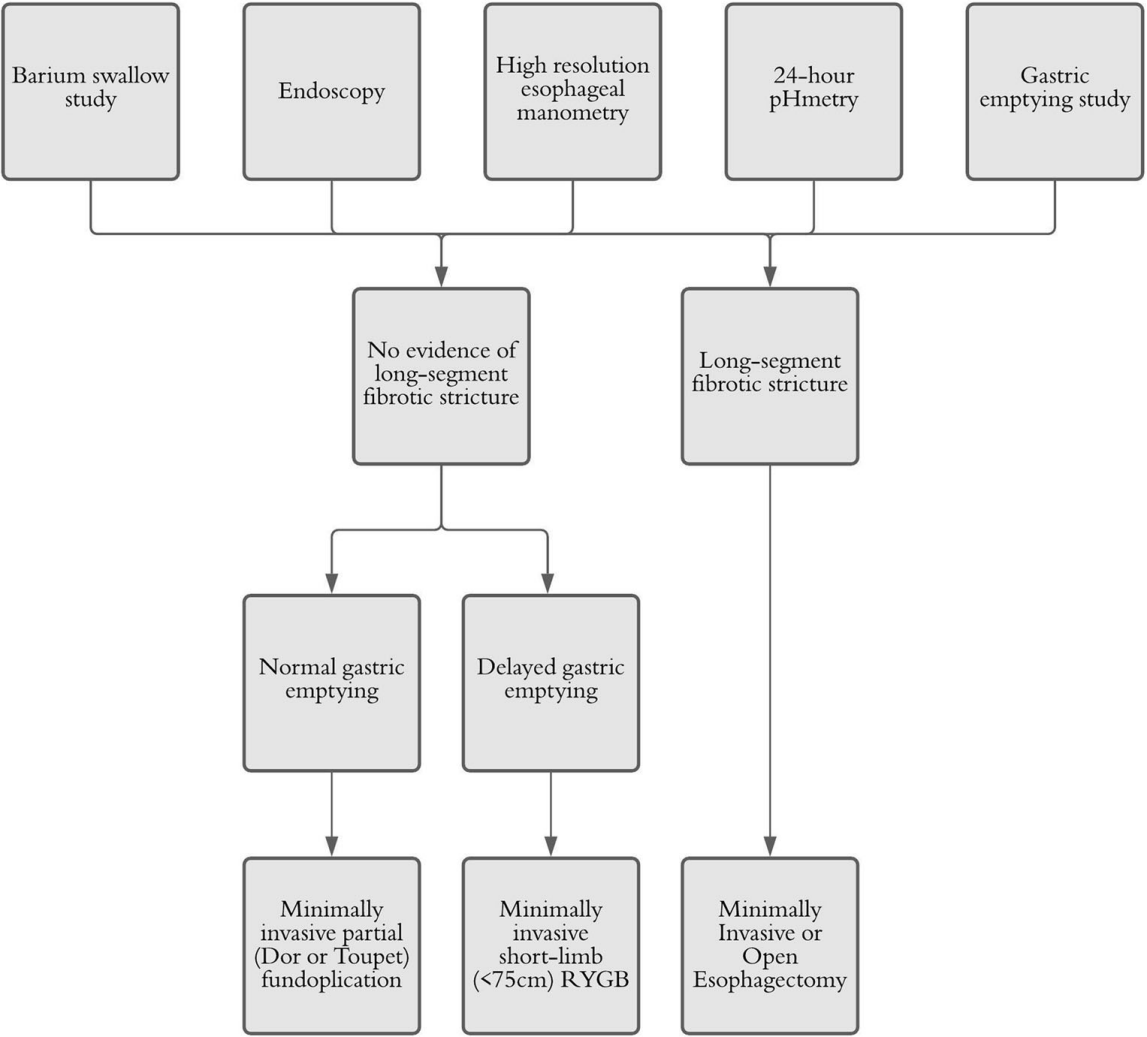
Proposed algorithm for the diagnosis and treatment of recalcitrant GERD in SSc patients

With recent advances in minimally invasive surgery, laparoscopic fundoplication has become the standard treatment in patients with symptomatic GERD and normal or impaired esophageal motility. Despite the concern that carbon dioxide insufflation in the abdominal cavity would not be well tolerated in patients with pulmonary fibrosis, the minimally invasive approach has gained increasing importance for the treatment of recalcitrant GERD in SSc patients. Goldberg and colleagues described results of 34 patients with GERD and esophageal hypomotility treated with laparoscopic fundoplication (Toupet, Dor, or Nissen) [20]. Overall, 38% of patients had a scleroderma-like esophagus and 10 were diagnosed with SSc. Improvement or resolution of GERD was recorded in 97% of patients with acceptable postoperative dysphagia rates. Similarly, Watson et al. described an effective GERD improvement in patients with aperistaltic esophagus and SSc with Dor and floppy Nissen fundoplication [21]. In an attempt to reduce the incidence of postoperative dysphagia and further improve reflux symptoms and esophagitis, two recent articles reported data for laparoscopic fundoplication and laparoscopic RYGB. Kent et al. described improved postoperative bloating symptoms (14% vs. 71%), diarrhea (14% vs. 43%), dysphagia (29% vs. 71%), and GERD-HRQL (4.0 vs. 15.6) for RYGB compared to fundoplication. Similarly, Yan et al. reported a complete reflux resolution in RYGB patients while 50% of patients in the fundoplication group reported unchanged symptoms. Furthermore, the authors suggested a potential benefit on pulmonary function, forced expiratory volumes in 1 s, and decreased immunosuppressant use after RYGB [17]. Because of the small number of patients, retrospective study design, different follow-up periods, and heterogeneous choice of fundoplication, it is not possible to determine whether RYGB is superior to fundoplication. Presumably, a short-limb RYGB may be beneficial in symptomatic SSc patients with delayed gastric emptying at the preoperative exams (barium swallow study and gastric scintigraphy). However, the limited follow-up, potentially associated small intestinal dysmotility disorders, restrictive/malabsorptive effects, and possibility of bacterial overgrowth should be considered. Given the heterogeneous indications, type of surgical procedure, and results, we propose a diagnostic and therapeutic algorithm for recalcitrant GERD in SSc patients (Fig. 2).

Laparoscopic fundoplication is a complex surgical procedure that requires a well-defined learning curve. The concept of Nissen fundoplication as the gold standard anti-reflux procedure has been challenged, especially in patients with impaired esophageal motility, with recent studies showing equivalent outcomes between Nissen and Toupet fundoplication [22–25]. Toupet fundoplication provides less outflow resistance with a better balance of reflux control, less dysphagia, gas bloat, and inability to belch [26]. Toupet fundoplication is not commonly performed and is technically more demanding compared to Nissen fundoplication [27]. However, a recently described standardized approach using a “critical-view” concept may improve reproducibility and clinical outcomes [28]. A complete distal esophageal mobilization is necessary to obtain an adequate intra-abdominal esophageal length (at least 2.5–3 cm). This may be useful to avoid esophageal lengthening procedure (Collis gastroplasty) that is indicated in exceptional cases (short esophagus) and should be pursued with caution in patients with severe motility disorders (http://www.oeso.org/OESO/books/Vol_3_Eso_Mucosa/Articles/ART164.HTML) [29]. On the other hand, anterior Dor fundoplication is easier and more standardized [30]. A recent meta-analysis of randomized controlled trials comparing Dor and Toupet fundoplication after Heller myotomy for esophageal achalasia showed comparable results in terms of postoperative abnormal acid exposure, % total time pH ≤ 4, mean DeMeester score, dysphagia, and basal LES pressure in the short-term follow-up [31]. However, in the setting of scleroderma, studies comparing outcomes for Dor and Toupet fundoplication are still lacking and warranted. Therefore, this review also aims to plea for further qualitative studies in order to codify the surgical procedure and better assess postoperative outcomes.

Major limitations of this systematic review are its small number of included patients, the possible background selection bias related to included studies’ heterogeneity, methodological quality, articles reporting, different treatments, anatomy of fundoplication, and surgeons’ experience. Patients were treated in different centers with diverse expertise and surgical skills. Finally, the choice of fundoplication was left to operating surgeons while surgical trends toward fundoplication over the last decades may constitute a further source of bias. Because SSc is a rare disease, it is challenging to perform a large prospective study and a systematic review may represent an attempt to give a comprehensive and updated analysis on such a debated topic.

## Conclusions

The management of SSc patients with GERD-related symptoms recalcitrant to medical treatment is challenging. Current literature is scarce and the data regarding appropriate surgical options are lacking. Esophagectomy should be reserved for selected patients with long-segment fibrotic stricture not amenable to endoscopic dilations. Minimally invasive RYGB appears feasible and safe with promising preliminary results in the short term while future studies are still needed to assess symptom remission, postoperative GERD, dysphagia, and pulmonary function in these patients. Well-designed studies are likewise needed to explore and better clarify feasibility, safety, and effectiveness of partial Dor and Toupet fundoplication.

### Supplementary information

ESM 1 (DOCX 13 kb).

## Data Availability

Data and datasets will be available upon reasonable request.

## Appendix

### Search strategy

#### PubMed/EMBASE/Web of Science

(Systemic Sclerosis OR Scleroderma) AND (gastroesophageal reflux disease OR GERD OR reflux) AND (antireflux surgery OR fundo* OR fundoplicatio OR fundoplication OR gastric fundoplication OR partial fundoplication OR Belsey OR Nissen OR Toupet OR Dor OR esophagectomy OR esophageal resection OR gastric bypass OR bypass) AND (thoracotomy OR laparotomy OR laparoscopy)
