# Supplementary material for: Surgical treatment of recalcitrant gastroesophageal reflux disease in patients with systemic sclerosis: a systematic review
Source: Langenbecks Arch Surg. 2021 Feb 21;406(5):1353–61. doi: 10.1007/s00423-021-02118-8 (PMC8370958; doi:10.1007/s00423-021-02118-8)
Supplement: Supplementary file 1 — (DOCX 13 kb). [file 423_2021_2118_MOESM1_ESM.docx]

| **Author** | **Confounding Bias** | **Selection Bias** | **Classification Bias** | **Intervention Bias** | **Missing Data Bias** | **Measurement Bias** | **Reporting Bias** | **Bias** |
| --- | --- | --- | --- | --- | --- | --- | --- | --- |
| Orringer et al, 1981 [12] | py | py | pn | pn | py | pn | pn | serious |
| Mansour et al, 1988 [13] | py | py | pn | pn | py | pyn | py | serious |
| Poirer et al, 1994 [14] | py | pn | pn | py | py | py | pn | serious |
| Kent et al, 2007 [8] | pn | pn | pn | pn | py | n | pn | moderate |
| Yekeler et al, 2008 [15] | py | py | pn | py | py | pn | pn | serious |
| Andrade et al, 2017 [16] | pn | py | py | py | py | n | py | moderate |
| Yan et al, 2018 [17] | pn | py | pn | py | pn | n | pn | moderate |

**Supplementary Table 1**. Quality assessment of the included studies (ROBINS-I tool). Each domain is evaluated with one of the following: y “yes”, py “probably yes”, pn “probably no”, and n “no”. The categories of judgement for each study are low, moderate, serious, and critical risk of bias.
